# Blood pressure management in hypertensive people with non-dialysis chronic kidney disease in Queensland, Australia

**DOI:** 10.1186/s12882-019-1532-6

**Published:** 2019-09-04

**Authors:** Jianzhen Zhang, Helen G. Healy, Sree Krishna Venuthurupalli, Ken-Soon Tan, Zaimin Wang, Anne Cameron, Wendy E. Hoy

**Affiliations:** 1 Level 8, Health Sciences Building, Building 16/901, Royal Brisbane & Women’s Hospitals, Herston, Brisbane, Queensland 4029 Australia; 2 Kidney Health Service (Royal Brisbane and Women’s Hospital), Metro North Hospital and Health Service, Brisbane, Queensland 4029 Australia; 3 Kidney Health Service (Toowoomba Hospital), Darling Downs Hospital and Health Service, Toowoomba, 4350 Queensland Australia; 4 Kidney Health Service (Logan Hospital), Metro South Hospital and Health Service, Logan, 4131 Queensland Australia

**Keywords:** Hypertension, Chronic kidney disease, Blood pressure control, Anti-hypertensive medications, Risk factors, CKD.QLD registry

## Abstract

**Background:**

High blood pressure is the most significant risk factor for the development and progression of chronic kidney disease (CKD). Lowering blood pressure is a goal to prevent CKD progression. This study of adults with CKD who have hypertension aimed to determine blood pressure control rates and the treatment patterns of hypertension and to explore factors associated with control of hypertension.

**Methods:**

This cross-sectional study included all non-dialysis people with CKD stages 3A to 5 under nephrology care in three public renal clinics in Queensland, who joined the CKD.QLD registry from May 2011 to Dec 2015 and had a history of hypertension. Demographic information, other health conditions, laboratory markers and anti-hypertensive medications in use at consent were extracted from the registry.

**Results:**

Among 1814 CKD people in these three sites in the registry who were age ≥ 18 years and had CKD stage 3A to 5, 1750 or 96% had a history of hypertension. Of these, the proportion with BP control to < 140/90 mmHg was 61.7% and to < 130/80 mmHg was 36.3%. With target BP < 140/90 mmHg or < 130/80 mmHg, participants aged ≥65 years were 1.23 (95% CI 1.06–1.42) or 1.12 (1.03–1.22) times more likely to have uncontrolled BP compared to those < 65 years old. Participants with severe albuminuria or proteinuria were 1.58 (1.32–1.87) or 1.28 (1.16–1.42, *p* < 0.001) more likely to have uncontrolled BP compared to those without significant albuminuria or proteinuria. Participants who had cardiovascular disease (CVD) were less likely to have uncontrolled BP compared to those without CVD (0.78, 0.69–0.89 or 0.86, 0.80–0.92). Factors associated with use of more classes of antihypertensive medicines among participants with uncontrolled BP (> 140/90 mmHg) were older age, diabetes, CVD, obesity and severe albuminuria/proteinuria (*p* < 0.05). Renin Angiotensin Aldosterone System inhibitors were the most frequently used medicines, regardless of the number of medicine classes an individual was prescribed.

**Conclusions:**

Blood pressure control rates in these hypertensive people with CKD was still far from optimal. People with CKD and hypertension aged 65 or older or with severe albuminuria or proteinuria, a group at risk of progression of kidney disease, have higher rates of uncontrolled BP.

## Background

Hypertension, also known as high blood pressure, is responsible for more deaths and disease worldwide than any other single health risk factor [1]. High blood pressure (BP) is the most significant risk factor for the development and progression of chronic kidney disease (CKD) [2, 3] and is present in up to 90% of individuals with CKD, with prevalence increasing as kidney function declines [4]. High BP can be a cause and/or a consequence of CKD, may develop early in the course of CKD and is associated with adverse outcomes such as worsening renal function and development of cardiovascular disease (CVD) [5]. High BP is an important risk factor for the development of CKD and a major leading cause of kidney failure after diabetes [6]. CKD shares many common risk factors with CVD and type 2 diabetes, such as overweight and obesity, physical inactivity, poor diet, tobacco smoking, and high blood pressure, all of which are potentially preventable [7]. Lowering blood pressure (BP) is a major treatment goal to prevent both CKD progression and CVD among people with CKD [8]. A recent meta-analysis study showed that intensive blood pressure lowering treatment significantly reduced risk of mortality among patients with CKD [9]. Optimal BP control is thus a major goal in preventing adverse outcomes in people with CKD.

The World Health Organization (WHO) defines high blood pressure as systolic blood pressure of ≥ 140 mmHg or diastolic blood pressure of ≥ 90 mmHg or receiving medication for high blood pressure [10]. Uncontrolled high blood pressure is defined as measured systolic blood pressure of 140 mmHg or more, or diastolic blood pressure of 90 mmHg or more, irrespective of the use of blood pressure medication. The KDIGO Guidelines recommend a target office BP in adults without albuminuria as consistently < 140/90 mmHg and in adults with any level of albuminuria as consistently < 130/80 mmHg irrespective of the presence of diabetes [11]. Controlling BP to target levels represents a therapeutic opportunity, but there is poor attainment of this goal, even amongst people with CKD receiving blood pressure medication [12, 13]. Early detection of high BP and its effective management to levels that are as close to target as possible makes a difference in the prevention of CKD progression and control of the CKD health burden [14]. At present, information on current practices in BP management among Australians with CKD is sparse.

In 2011, we established the CKD.QLD Registry for the surveillance, practice improvement and research of CKD. The CKD.QLD Registry, which has been described elsewhere [15], is a network of nephrology practices in the public health system in Queensland. This study aims to determine blood pressure control rates, the treatment patterns of hypertension and risk factors for uncontrolled blood pressure in hypertensive adults with pre-terminal (non-dialysis) chronic kidney disease in this registry.

## Methods

### Study design and setting

This cross sectional study includes all patients from three major public renal services in Queensland who gave consent to join the CKD.QLD registry from May 2011 to Dec 2015 and had complete records of BP readings and anti-hypertensive medications at time of consent. We defined hypertension as a recorded co-morbidity in the medical records and/or prescription of anti-hypertensive medications recorded when participants consented to join the Registry.

### Study participants

Recruitment into the CKD.QLD registry is described elsewhere [16]. Briefly, participants over the age of 18 years who are referred to Queensland Health renal services and are not on renal replacement therapy (RRT) are offered the opportunity to join the CKD.QLD Registry and those who do so are followed until they reach one of the outcomes of either death, or RRT, or by specified censor date.

The present study selected adult (≥18 years) participants who met the inclusion criteria of a history of hypertension at baseline and who had CKD stage 3A to 5, who were under public nephrology care from three main study sites in the CKD.QLD Registry. Patients age < 18 years and those receiving dialysis or with a kidney transplant were excluded.

### Study measurements

Participant demographic information (age group, sex, and indigenous status), health conditions (history of CVD, diabetes, and obesity), smoking status, laboratory markers [estimated glomerular filtration rate (eGFR), albuminuria or proteinuria (ACR/PCR) categories] and anti-hypertensive medications were extracted from the CKD.QLD registry. A history of CVD is a binary variable derived from the patient record. If the participants had coronary artery disease, prior revascularisation, heart failure, stroke or peripheral vascular diseases, then they assigned as having had cardiovascular disease. Diabetes is a binary variable indicating the history of diabetes at baseline. It includes serum fasting glucose ≥7.0 mmol/L (≥126 mg/dL), non-fasting glucose ≥11.1 mmol/L (≥200 mg/dL), glycated hemoglobin A1c ≥6.5%, and/or use of glucose lowering drugs, and is also derived from a renal diagnosis of diabetic nephropathy +/− a comorbidity of diabetes. Obesity is a binary variable indicating whether BMI was < 30 kg/m^2^ or ≥ 30 kg/m^2^. Smoking status was recorded as dichotomous variable (yes/no) indicating whether the participant currently smoked cigarettes or not. The four groups of eGFR were CKD stage 3A (45 ≤ eGFR < 60), 3B (30 ≤ eGFR < 45) and 4 (≥15 to < 30) and 5(< 15). The three groups of ACR/PCR were normal (ACR < 30 mg/g or PCR < 150 mg/g), mild (30 ≤ ACR < 300 mg/g or 150 ≤ PCR < 500 mg/g) and severe (ACR ≥300 mg/g or PCR ≥500 mg/g).

We stratified the study population blood pressures as uncontrolled versus controlled, where lack of control was defined by either of two international targets. The first is the WHO definition of measured systolic blood pressure (SBP) of ≥140 mmHg, and/or diastolic blood pressure (DBP) of ≥90 mmHg. The second is the more complex KDIGO guideline for blood pressure control for kidney disease taking into account albuminuria and consists of measured SBP ≥130 mmHg, and/or DBP ≥80 mmHg [11, 17, 18].

We evaluated patterns of drug use firstly by the number of anti-hypertensive drug classes, as defined by the Australian Medicines Handbook [17]. Medications were categorised into Renin Angiotensin Aldosterone System (RAAS) inhibitors (angiotensin-converting-enzyme or CE inhibitors and angiotensin II receptor blockers, or ARBs), diuretics, calcium channel blockers (CCB), beta-blockers and/or other classes (Clonidine, Diazoxide, Hydralazine, Methyldopa, Minoxidil, Moxonidine, and Sodium nitroprusside).

### Statistical analysis

We compared participant characteristics at consent according to BP control status at the two target levels. Bivariate relationships between BP control and each of the variables of interest, and number of anti-hypertensive drug classes and each of the variables of interest were explored using Chi-square analyses. Generalised linear models (GLMs), with binomial distribution and log link [19], were performed to estimate adjusted prevalence ratios of uncontrolled BP for each individual variable.

Multivariable modelling was performed to explore the factors associated with BP control at two levels, including age, sex, smoking, other health conditions (CVD, diabetes, and obesity) and laboratory markers (eGFR and ACR/PCR). Due to the small numbers, indigenous status was not included in the models. There were also missing data in smoking status, obesity and ACR/PCR category. Only participants with the complete set of covariates were included in the multivariable analyses. In addition, multivariable modelling was performed to examine the prevalence ratio of uncontrolled BP according to anti-hypertensive drug classes adjusting for age and sex.

Preliminary adjusted GLMs [20] tested the feasibility of analysing multiple subgroups of each variable. We found sample size in some subgroups precluded the statistical test and therefore transformed age from three groups (≥18 to < 45, ≥45 to 65, and ≥ 65) into two (< 65 vs ≥65), and eGFR from four groups (≥45 to < 60, ≥30 to < 45, ≥15 to < 30 and < 15) into two (< 30 vs ≥30). A reference group for each categorical variable was defined in GLMs. When a binary variable represents the presence versus absence of a health condition, the group without the condition was used as the reference group, for example, non-CVD, non-diabetes and non-obesity were the reference group for CVD, diabetes and obesity respectively. When a categorical variable represented more than two groups, such as age group, eGFR and ACR/PCR category, the group at the first level was used as the reference group, e.g. age < 65, eGFR ≥30, and normal ACR/PCR were the reference group respectively. When exploring the effect of sex and indigenous status, male and non-indigenous were the reference group respectively. All analyses were undertaken using Stata 14.1 (Stata Corp. Stata Statistical Software: Release 14.1, College Station. TX: StatCorp LP, 2016). Statistical significance is defined as a *p* value < 0.05 (two-tail).

## Results

### Participant characteristics

Of the 1814 age- and CKD stage-eligible participants from these three sites enrolled in CKD.QLD at the time of this study, 1750, or 96%, had a history of hypertension. Table 1 presents BP control status to the two target levels according to patient characteristics. The majority (72.2%) were aged 65+ years, 52.5% were males, only 3.7% were indigenous, 53.6% had diabetes, 56.2% had CVD and 38.2% had advanced CKD (31.5% with stage 4 and 6.7% with stage 5) with an eGFR less than 30 ml/min/1.73 m^2^ body surface area. Of 1719 participants with available data, 31.9 and 40.3% had mildly or severely elevated levels of ACR/PCR.

**Table 1:** BP control status at 140/90 mmHg and 130/80 mmHg according to patient characteristics

Characteristics	Controlled BP < 140/90^a^		*P*-Value^b^	Controlled BP < 130/80^a^		*P*-Value^b^	Overall^c^
Overall	Yes (*n* = 1080)	No (*n* = 670)		Yes (*n* = 635)	No (*n* = 1115)		*N* = 1750
Age group			0.402			0.891	
≥ 18 to < 45	53 (57.6)	39 (42.4)		33 (35.9)	59 (64.1)		92 (5.3)
≥ 45 to < 65	253 (64.2)	141 (35.8)		147 (37.3)	247 (62.7)		394 (22.5)
≥ 65	774 (61.2)	490 (38.8)		455 (36.0)	809 (64.0)		1264 (72.2)
Sex			0.633			0.399	
Male	572 (62.2)	347 (37.8)		325 (35.4)	594 (64.6)		919 (52.5)
Female	508 (61.1)	323 (38.9)		310 (37.3)	521 (62.7)		831 (47.5)
Indigenous (*n* = 1368)			0.132			0.137	
Yes	25 (50.0)	25 (50.0)		13 (26.0)	37 (74.0)		50 (3.7)
No	799 (60.6)	519 (39.4)		478 (36.3)	840 (63.7)		1318 (96.4)
Smoking (*n* = 1599)			0.149			0.365	
Yes	88 (66.7)	44 (33.3)		52 (39.4)	80 (60.6)		132 (8.3)
No	884 (60.3)	583 (39.7)		520 (35.4)	947 (64.6)		1467 (91.7)
Diabetes			0.273			0.389	
Yes	590 (62.9)	348 (37.1)		349 (37.2)	589 (62.8)		938 (53.6)
No	490 (60.3)	322 (39.7)		286 (35.2)	526 (64.8)		812 (46.4)
CVD			**0.001**			**0.001**	
Yes	647 (65.8)	337 (34.2)		399 (40.5)	585 (59.5)		984 (56.2)
No	433 (56.5)	333 (43.5)		236 (30.8)	530 (69.2)		766 (43.8)
Obesity (*n* = 1729)			0.824			0.927	
Yes	546 (62.3)	331 (37.7)		322 (36.7)	555 (63.3)		877 (50.7)
No	526 (61.7)	326 (38.3)		311 (36.5)	541 (63.5)		852 (49.3)
eGFR			**0.013**			0.067	
≥ 45 to < 60	240 (66.5)	121 (33.5)		152 (42.1)	209 (57.9)		361 (20.6)
≥ 30 to < 45	448 (62.2)	272 (37.8)		255 (35.4)	465 (64.6)		720 (41.1)
≥15 to < 30	333 (60.4)	218 (39.6)		190 (34.5)	361 (65.5)		551 (31.5)
< 15	59 (50.0)	59 (50.0)		38 (32.2)	80 (67.8)		118 (6.7)
ACR/PCR (*n* = 1719)			**0.001**			**0.001**	
Normal	337 (70.5)	141 (29.5)		209 (43.7)	269 (56.3)		478 (27.8)
Mild	347 (63.2)	202 (36.8)		206 (37.5)	343 (62.5)		549 (31.9)
Severe	379 (54.8)	313 (45.2)		208 (30.1)	484 (69.9)		692 (40.3)
# drug classes			**0.008**			0.105	
0	36 (47.4)	40 (52.6)		23 (30.3)	53 (69.7)		76 (4.3)
1	270 (67.7)	129 (32.3)		150 (37.6)	249 (62.4)		399 (22.8)
2	311 (61.8)	192 (38.2)		176 (35.0)	327 (65.0)		503 (28.7)
3	288 (60.8)	186 (39.2)		191 (40.3)	283 (59.7)		474 (27.1)
≥ 4	175 (58.7)	123 (41.3)		95 (31.9)	203 (68.1)		298 (17.0)
Drug classes							
RAAS inhibitors	763 (63.1)	446 (36.9)	0.073	456 (37.7)	753 (62.3)	0.063	1209 (69.1)
-- ACE inhibitors^d^	450 (62.8)	267 (37.2)	0.453	278 (38.8)	439 (61.2)	0.071	717 (41.0)
-- ARBs^d^	360 (62.6)	215 (37.4)	0.590	204 (35.5)	371 (64.5)	0.623	575 (32.9)
Beta-blockers	538 (64.0)	303 (36.0)	0.062	334 (39.7)	507 (60.3)	**0.004**	841 (48.1)
CCB	477 (57.3)	355 (42.7)	**0.001**	258 (31.0)	574 (69.0)	**0.001**	832 (47.5)
Diuretics	503 (63.0)	295 (37.0)	0.299	308 (38.6)	490 (61.4)	0.066	798 (45.6)
Other classes	168 (50.9)	162 (49.1)	**0.001**	96 (29.1)	234 (70.9)	**0.003**	330 (18.9)

^a^Row percentage presented

^b^Chi-square tests on relationships between BP control and each of the variables of interest

^c^Column percentage presented

^d^Sub-class of RASS inhibitors

Significant values (*p*<0.05) were in bold entries

### BP control rates

Overall, 61.7 and 36.3% of participants had BP controlled to SBP/DBP < 140/90 and < 130/80 mmHg respectively at time of enrolment to the registry.

Bivariate analyses show that BP control to SBP/DBP < 140/90 or < 130/80 mmHg in participants without CVD (56.5% or 30.8%), or with eGFR < 15 (50.0% or 32.2%), or with severe ACR/PCR elevations (54.8 and 30.1%) was worse than the respective referent groups (all *p* < 0.05 except eGFR< 15 with BP control < 130/80 as *p* = 0.067), irrespective of which method was used to define target. Table 1 also shows that there were no statistical differences in terms of BP control among age groups, or by sex, indigenous status, smoking status, diabetes and obesity (*p* > 0.05).

### Anti-hypertensive medications

Table 1 also shows the distribution of use of the major classes of current antihypertensive medications recorded in patients’ clinical records, noting that > 70% of participants were using more than one class. Roughly equal numbers of participants used one (22.8%), two (28.7%) or three (27.1%) classes of anti-hypertensive medications, and 17% were using four or more drug classes. Only 4.3% did not use any anti-hypertensive medications. Of the participants using one (a single) anti-hypertensive drug class, 67.7% achieved BP control to the < 140/90 target, which was a higher rate of control than those using more than one class of drug (58.7–61.8%) (*p* < 0.01). There were no differences in the number of drug classes used to control BP to WHO compared to KDIGO targets. Rates of control of BP were lower in groups prescribed more classes of antihypertensive drugs.

**Fig. 1:**
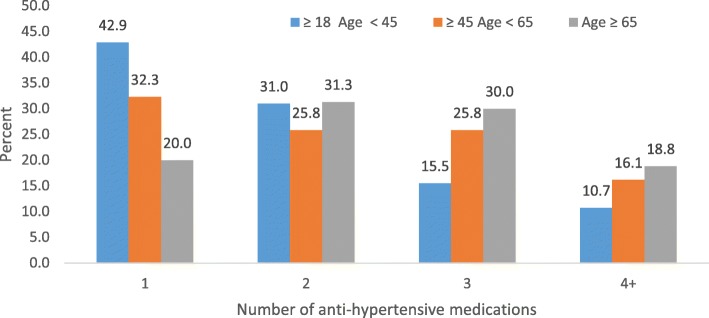
Percentage of anti-hypertensive medications by age group

Among all participants, RAAS inhibitors were the most frequently prescribed medication group (69.1% of participants), followed by beta-blockers and CCB (48% of participants each) and diuretics (45.6% of participants). Mineralocorticoid receptor antagonists, included as diuretics, were used in 3.2% of the cohort (or 7% of those on diuretics). Table 2 shows the number of anti-hypertensive drugs used by participants with uncontrolled BP (≥140/90 mmHg) by age groups, health conditions and laboratory markers. Older participants (≥ 65 years) were using more classes of drugs i.e. two, three and four or more anti-hypertensive drugs (*p* < 0.001) (Fig. 1). Participants with diabetes, CVD, obesity or severe ACR/PCR also used more anti-hypertensive drug classes (*p* < 0.05). There was no significant difference among eGFR group in terms of number of drug class use (*p* = 0.138).

**Table 2:** Number of anti-hypertensive drug classes among patients with uncontrolled BP (≥140/90 mmHg) according to age groups, health conditions and laboratory markers

Variables	N	Number of anti-hypertensive drug classes					*P*-value
		0	1	2	3	4 +	
Age group (years)							
≥ 18 to < 45	39	5 (12.8)	14 (35.9)	11 (28.2)	4 (10.3)	5 (12.8)	**0.001**
≥ 45 to 65	141	11 (7.8)	38 (27.0)	28 (19.9)	40 (28.4)	24 (17.0)	
≥ 65	490	24 (4.9)	77(15.7)	153 (31.2)	142 (29.0)	94 (19.2)	
Diabetes							
Yes	348	8 (2.3)	57 (16.4)	97 (27.9)	108 (31.0)	78 (22.4)	**0.001**
No	322	32 (9.9)	72 (22.4)	95 (29.5)	78 (24.2)	45 (14.0)	
Obesity							
Yes	331	10 (3.0)	63 (19.3)	93 (28.1)	97 (29.3)	68 (20.5)	**0.014**
No	326	30 (9.2)	63 (19.3)	95 (29.1)	84 (25.8)	54 (16.6)	
CVD							
Yes	337	14 (4.2)	45 (13.4)	98 (29.1)	105 (31.2)	75 (22.3)	**0.001**
No	333	26 (7.8)	84 (25.2)	94 (28.2)	81 (24.3)	48 (14.4)	
eGFR							
≥ 45 to < 60	121	10 (8.3)	32 (26.5)	37 (30.6)	24 (19.8)	18 (14.9)	0.138
≥ 30 to < 45	272	16 (5.9)	50 (18.4)	85 (31.3)	75 (27.6)	46 (16.9)	
≥ 15 to < 30	277	11 (5.1)	33 (15.1)	59 (27.1)	70 (32.1)	45 (20.6)	
< 15		3 (5.1)	14 (23.7)	11 (18.6)	17 (28.8)	14 (23.7)	
ACR/PCR categories							
Normal	141	7 (5.0)	34 (24.1)	47 (33.3)	37 (26.2)	16 (11.4)	**0.042**
Mild	202	18 (8.9)	36 (17.8)	61 (30.2)	53 (26.2)	34 (16.8)	
Severe	313	13 (4.2)	57 (18.2)	84 (26.8)	88 (28.1)	71 (22.7)	

Significant values (*p*<0.05) were in bold entries

**Fig. 2:**
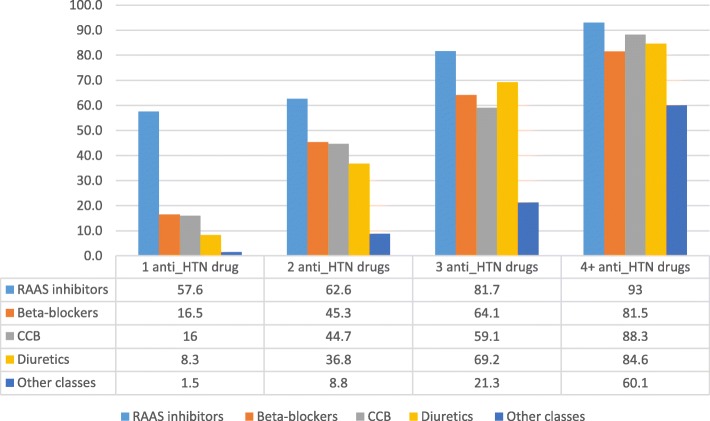
The distribution of type of anti-hypertensive medications by the number of medicine classes

Figure 2 shows the distribution and frequency of anti-hypertensive drugs by number of drug classes. RAAS inhibiting drugs (ACEI and/or ARBs) were the most frequently used categories of medicine, regardless of the number of medicine classes that an individual was prescribed. They were included in the treatment regimen of 57.6, 62.6, 81.7% of people receiving one, two and three drug classes respectively, and of 93% of participants receiving four or more drug classes. The next most frequently prescribed medicines were beta-blockers (16.5, 45.3, 64.1, and 81.5%), calcium channel blockers (16, 44.7, 59.1, and 88.3%) and diuretics (8.3, 36.8, 69.2, and 84.6%) from one to four or more drug use. Other classes of anti-hypertensive drugs were infrequently applied except in subjects requiring four or more classes of medicine. As participant’s usage of anti-hypertensive drug classes increased, the pattern became more homogenous i.e. all drugs classes increased when participants used four drug classes.

Table 3 presents the prevalence ratio of uncontrolled BP according to anti-hypertensive drug classes adjusting for age and sex. It suggests that beta-blockers were associated with a lesser frequency of BP uncontrolled at the 130/80 mmHg target and this relationship is statistically significant (0.90, 0.83–0.97). RAAS inhibitors were also associated with less BP uncontrolled to 140/90 mmHg (0.92, 0.81–1.04), but it is not statistically significant (*p* = 0.192), and to 130/80 mmHg (0.93, 0.86–1.00), which is a bottom line statistically significant (*p* = 0.049). CCB and other anti-hypertensive drugs were significantly associated with more likelihood of uncontrolled BP to 140/90 mmHg (CCB: 1.22, 1.07–1.39; other classes: 1.26, 1.09–1.45), and to 130/80 mmHg (CCB: 1.16, 1.08–1.25; other classes: 1.11, 1.02–1.21).

**Table 3:** Multivariable prevalence ratio of uncontrolled BP according to anti-hypertensive drug classes (*n* = 1569)

	Uncontrolled BP (≥140/90)			Uncontrolled BP (≥130/80)		
	PR	95% CI	*P*-Value	PR	95% CI	*P*-Value
Age ≥ 65	1.11	0.96–1.27	0.177	1.05	0.97–1.14	0.222
Female	1.04	0.92–1.17	0.548	0.97	0.90–1.04	0.357
RAAS inhibitors	0.92	0.81–1.04	0.192	0.93	0.86–1.00	0.049
Beta-blockers	0.91	0.80–1.03	0.135	0.90	0.83–0.97	**0.004**
Diuretics	0.92	0.82–1.05	0.223	0.93	0.87–1.01	0.072
CCB	1.22	1.07–1.39	**0.002**	1.16	1.08–1.25	**0.001**
Other classes	1.26	1.09–1.45	**0.002**	1.11	1.02–1.21	**0.013**

Significant values (*p*<0.05) were in bold entries

### Risk factors and BP control

Table 4 presents the multivariable adjusted model of prevalence ratio of uncontrolled BP, analysed to both targets, exploring the effects of age, sex, smoking, obesity, diabetes, history of CVD, eGFR and ACR/PCR. Taking < 140/90 mmHg BP as the target, participants aged ≥65 years were 1.23 times more likely to have uncontrolled BP (1.06–1.42) compared those < 65 years old (*p* < 0.01). Participants with CVD were less likely to have uncontrolled BP (0.78, 0.69–0.89) compared to those without CVD (*p* < 0.001). Participants with severe ACR/PCR elevations were 1.58 (1.32–1.87, *p* < 0.001) more likely to have uncontrolled BP to < 140/90 mmHg compared to those without significant ACR/PCR.

**Table 4:** Multivariable prevalence ratio of uncontrolled BP according to age group, sex, smoke, diabetes, history of CVD, obesity, eGFR, ACR/PCR (*n* = 1569)

	No.	Uncontrolled BP (≥140/90)			Uncontrolled BP (≥130/80)		
		PR	95% CI	*P*-Value	PR	95% CI	*P*-Value
Age group							
Age < 65	446 (28.4)	1.00 (Ref)			1.00 (Ref)		
Age ≥ 65	1123 (71.6)	1.23	1.06–1.42	**0.006**	1.12	1.03–1.22	**0.009**
Sex							
Male	815 (51.9)	1.00 (Ref)			1.00 (Ref)		
Female	754 (48.1)	1.02	0.90–1.15	0.735	0.95	0.89–1.03	0.198
Smoke							
No	1438 (91.7)	1.00 (Ref)			1.00 (Ref)		
Yes	131 (8.4)	1.12	0.87–1.43	0.387	1.04	0.90–1.20	0.575
Diabetes							
No	735 (46.9)	1.0 0(Ref)			1.00 (Ref)		
Yes	834 (53.2)	0.92	0.81–1.05	0.22	0.98	0.91–1.06	0.614
CVD							
No	694 (44.2)	1.00 (Ref)			1.00 (Ref)		
Yes	875 (55.8)	0.78	0.69–0.89	**0.001**	0.86	0.80–0.92	**0.001**
Obesity							
No	769 (49.0)	1.00 (Ref)			1.00 (Ref)		
Yes	800 (51.0)	1.06	0.93–1.20	0.391	1.00	0.93–1.08	0.867
eGFR							
eGFR ≥30	971 (61.9)	1.00 (Ref)			1.00 (Ref)		
eGFR < 30	598 (38.1)	0.99	0.87–1.12	0.86	0.96	0.89–1.05	0.423
ACR/PCR							
Normal	431 (27.5)	1.00 (Ref)			1.00 (Ref)		
Mild	503 (32.1)	1.19	0.99–1.43	0.066	1.11	1.00–1.24	0.054
Severe	635 (40.5)	1.58	1.32–1.87	**0.001**	1.28	1.16–1.42	**0.001**

Significant values (*p*<0.05) were in bold entries

The associations of these variables with BP control was similar when the target was 130/80 mmHg. Participants aged ≥65 years were 1.12 times more likely to have uncontrolled BP (1.03–1.22) compared those < 65 years old (*p* < 0.01). Participants who had CVD were less likely to have uncontrolled BP (0.86, 0.80–0.92) compared to those without CVD (*p* < 0.001). Participants with severe ACR/PCR elevations were 1.28 (1.16–1.42, *p* < 0.001) more likely to have uncontrolled BP compared to normal group.

## Discussion

This study of 1750 hypertensive participants with non-dialysis CKD found that the prevalence of BP control to the WHO target of < 140/90 mmHg was 61.7% and to the KDIGO target of < 130/80 mmHg was 36.3%. Thus there is opportunity to optimise this powerful risk factor in a population that already has CKD.

Older participants (aged ≥65 years) were more likely to have uncontrolled BP at both targets of 140/90 mmHg and 130/80 mmHg compared to those aged< 65 years, which is consistent with the literature [6, 13]. For many years, there have been concerns that old age is a barrier to the treatment of hypertension due to “potential poor tolerability, and even harmful effects of BP-lowering interventions”. However, evidence from RCTs shows that antihypertensive treatment substantially reduces CV morbidity and CV and all-cause mortality in old and very old patients [21, 22]. Notwithstanding the systematic exclusion of the elderly and extreme elderly from interventional drug trials, the HYVET study reported that these age groups also benefit from lowering SBP to at least < 150 mmHg [21]. Participants with severe ACR/PCR elevations were more likely to have uncontrolled BP at both targets compared to those without significant elevations in ACR/PCR. Koroshi proposes that high blood pressure may cause microalbuminuria by increasing glomerular filtration pressure and subsequent renal damage [23] and other data have linked BP control to a reduction in urine albumin levels [24]. The effect of BP control may be mediated through albuminuria or proteinuria, and Mani [24] has reported that BP control without improvement in albuminuria/proteinuria did not have an adequate effect in slowing the progression of CKD. Many studies also show that that albuminuria or proteinuria are strong and independent predictors of the risks of CKD progression, CVD and all-cause mortality [25–29]. Therefore, screening for microalbuminuria in everyone with hypertension has been recommended [30].

In this cohort, and in contrast to a previous study [14], participants who had CVD were less likely to have uncontrolled BP compared to those without CVD. Perhaps this reflects awareness of service providers that higher BP is a risk factor for CVD and a concentrated effort to control BP in people already known to have CVD complications. The Australian federal government has targeted chronic diseases such as CVD, diabetes and obesity as priority areas and has set specific strategies and benchmarks for the management of risk factors for, and the prevention of, these chronic diseases, demonstrating the public policy approach [31].

The group in our study population with uncontrolled BP tended to be using more classes of anti-hypertensive drugs, suggesting that a stepped care or multidrug response has been taken to difficult-to-management hypertension. They were also older (≥65 years) and more often had diabetes, CVD, obesity and severe ACR/PCR elevations. The highest prevalence of hypertension control was in participants on only one class of antihypertensive medications. This group also had less CVD co-morbidity (39.6%), less diabetes (43.1%) and better kidney function, with eGFR> 30 in 69.7%. The most common first line drug was either an ACE inhibitor (35.3%) or ARB (22.3%). We are unable to determine whether the drug was initiated for treatment of proteinuria rather than hypertension or for both reasons.

Physicians appeared at least partly to be following international recommendations for the management of CKD. RAAS inhibitors (ACE inhibitors and ARBs) were the most frequently used medications, in line with their reported renoprotective effect in addition to their anti-hypertensive properties. Where only one anti-hypertensive drug was listed, an ACE inhibitor was the most common class, followed by ARBs. The efficacy of these drugs is well established [18]. However, this pattern of anti-hypertensive drugs classes changed as additional drugs were added. When two anti-hypertensive drugs were used, beta blockers and CCB were the most common classes, followed by diuretics and only then ACE inhibitors. It is not clear why physicians diverge from the recommendations of guidelines in those cases where BP control, as measured by the surrogate of number of drug classes, becomes more difficult. It is likely that a proportion of participants did not respond to, or did not tolerate, the RAAS group drugs. There are scant data in the literature of BP control, particularly when more than two drugs are prescribed. In the most difficult cases, the evidence base is non-existent, and all drugs classes are used.

Our study is one of the few examining BP management in real world people with non-dialysis CKD. Data were derived from three main study sites across Queensland and the enrolment representation of patients with CKD in these three sites at that time was 55, 86 and 95% respectively.

We acknowledge limitations in this study. The study sampled BP management at one time point, when patients consented to the CKD.QLD registry, and we know that BP varies over time and according to the measurement environment. There is the risk of mislabelling [32]. The selection of sites into this study may not reflect clinical practice in all specialty nephrology settings in Queensland. Sample size was inadequate to study separately some important groups, like indigenous people, and it limited some comparisons. Furthermore, we analysed the medications based on the assumption that the medications prescribed were really taken by the patients. There is a major issue of non-compliance when multiple anti-hypertensive drugs are prescribed.

The WHO has established a specific target to reduce prevalence of elevated BP (≥140/90 mmHg) by 25% by 2025 and has recommended that healthcare professionals measure BP at all relevant clinical encounters [33]. The World Hypertension League (WHL) expert committee also recommended a set of standardised performance indicators to improve BP control at both the population and healthcare organisation levels [34]. The National Heart Foundation of Australia produced and updated “Guidelines for the diagnosis and management of hypertension in adults” for the information of health professionals in 2016 [35]. These guidelines have been endorsed by Kidney Health Australia, the National Stroke Foundation and the High Blood Pressure Research Council of Australia. The Royal Australian College of General Practitioners has recommended the Guideline for approval as an Accepted Clinical Resource [36]. There are strong recommendations for treatment strategies and treatment targets for people with hypertension, representing strong public policy approaches. Despite these, controlling elevated blood pressure in individual people and at a population level remains a large national challenge.

## Conclusions

Our findings show considerable success in achieving BP targets set by international guidelines in people with CKD. However, we must acknowledge that BP control does not meet international benchmarks yet. The data also reveal variations in the patterns of hypertension management. Health care providers commonly used several classes of antihypertensive drugs, and, appropriately, used more classes of anti-hypertensive drugs in those who had uncontrolled BP. Factors associated with use of more classes of antihypertensive medicines among people with uncontrolled BP (> 140/90 mmHg) were older age, diabetes, CVD, obesity and severe albuminuria/proteinuria. We also found additional risk factors for poor BP control, such as older age (≥ 65 years) and/or severe ACR/PCR elevations. Better BP control has important benefits for people with CKD. As evidenced, salt restriction also improves BP control in many patients with CKD (Stage 1–4) [37]. Therefore, reducing the main risk factors can be achieved through a moderate reduction of salt intake. However, there is some room for more pervasive BP control in this population of difficult and complex people.

## Data Availability

The dataset used for this current study are available from the corresponding author on reasonable request.

## Abbreviations

ACE: Angiotensin-converting-enzyme
ACR/PCR: Albuminuria or Proteinuria
ARBs: Angiotensin II receptor blockers
BP: Blood pressure
CCB: Calcium channel blockers
CKD.CRE: Chronic kidney disease centre of research excellence
CKD.QLD: Chronic kidney disease, Queensland
CVD: Cardiovascular disease
DBP: Diastolic blood pressure
eGFR: Estimated Glomerular Filtration Rate
RAAS: Renin angiotensin aldosterone System
RRT: Renal replacement therapy
SBP: Systolic blood pressure
WHO: World Health Organization
